# E-Nose: Time–Frequency Attention Convolutional Neural Network for Gas Classification and Concentration Prediction

**DOI:** 10.3390/s24134126

**Published:** 2024-06-25

**Authors:** Minglv Jiang, Na Li, Mingyong Li, Zhou Wang, Yuan Tian, Kaiyan Peng, Haoran Sheng, Haoyu Li, Qiang Li

**Affiliations:** 1 Key Laboratory of Physical Electronics and Devices for Ministry of Education and Shaanxi Provincial Key Laboratory of Photonics & Information Technology, Xi’an Jiaotong University, Xi’an 710049, China; jml.vito@stu.xjtu.edu.cn; 2 School of Electronic Science and Engineering, Xi’an Jiaotong University, Xi’an 710049, China; pkymail@stu.xjtu.edu.cn (K.P.); baekhyun_shr_2001@stu.xjtu.edn.cn (H.S.); msn010@stu.xjtu.edu.cn (H.L.); 3 Northwest Survey & Planning Institute of National Forestry and Grassland Administration, Xi’an 710048, China; superman1232004@126.com (N.L.); xby107@163.com (Z.W.); 4 Key Laboratory of National Forestry and Grassland Administration on Ecological Hydrology and Disaster Prevention in Arid Regions, Xi’an 710048, China; 5 CSSC AlphaPec Instrument (Hubei) Co., Ltd., Yichang 443005, China; limingyong0430@163.com; 6 China National Engineering Laboratory for Coal Mining Machinery, CCTEG Taiyuan Research Institute Co., Ltd., Taiyuan 030032, China; 13643462056@139.com

**Keywords:** electronic nose, gas sensor, time–frequency attention, convolutional neural network

## Abstract

In the electronic nose (E-nose) systems, gas type recognition and accurate concentration prediction are some of the most challenging issues. This study introduced an innovative pattern recognition method of time–frequency attention convolutional neural network (TFA-CNN). A time–frequency attention block was designed in the network, aiming to excavate and effectively integrate the temporal and frequency domain information in the E-nose signals to enhance the performance of gas classification and concentration prediction tasks. Additionally, a novel data augmentation strategy was developed, manipulating the feature channels and time dimensions to reduce the interference of sensor drift and redundant information, thereby enhancing the model’s robustness and adaptability. Utilizing two types of metal-oxide-semiconductor gas sensors, this research conducted qualitative and quantitative analysis on five target gases. The evaluation results showed that the classification accuracy could reach 100%, and the coefficient of the determination (*R*^2^) score of the regression task was up to 0.99. The Pearson correlation coefficient (*r*) was 0.99, and the mean absolute error (MAE) was 1.54 ppm. The experimental test results were almost consistent with the system predictions, and the MAE was 1.39 ppm. This study provides a method of network learning that combines time–frequency domain information, exhibiting high performance in gas classification and concentration prediction within the E-nose system.

## 1. Introduction

E-nose systems, often regarded as artificial olfaction systems, primarily consist of a series of sensors sensitive to specific gases and pattern recognition algorithms. In an E-nose, the perception of odors starts with gas sensors. Similar to how gas molecules bind to receptors on olfactory neurons in the sense of smell, gas molecules in the air interact with the sensing material within gas sensors, leading to measurable changes in electronic signals. These signals are then processed by neural network algorithms. Neural networks fragment the information through connections of neural nodes and link these fragments to extract and process more complex (abstract) information, and finally get the result of the analysis. This technology, capable of mimicking biological olfaction mechanisms, has become an economical and efficient non-destructive testing technique, widely applied in fields such as biomedical diagnosis [1], industrial emission monitoring [2], and food quality assessment [3]. Given this, enhancing the detection capability and resolution of electronic nose systems is crucial. There are two basic ways to improve the performance of E-nose systems. One is to increase the sensitivity and selectivity of gas sensors by improving the sensitive materials, device structure, and manufacturing process [4,5,6,7,8]. The other approach involves employing advanced signal pre-processing methods, new software tools, and new algorithms [9,10,11,12,13] to improve the analytical performance of the pattern recognition algorithms.

In E-nose systems, sensor signals are typically represented as one-dimensional or multidimensional time series. These signals intuitively reflect the dynamic changes of specific attributes over time through their amplitudes, hence their widespread use. Although this effectively reveals the intuitive characteristics of sensor signals, current research on feature extraction in the frequency domain is relatively limited. Strategies primarily focused on time domain feature extraction may not fully explore the comprehensive information within signals. Frequency domain analysis offers a frequency-based perspective on signals, revealing their deeper characteristics and thus complementing the inadequacies of time domain analysis, involving a variety of methods such as Fourier transform, discrete cosine transform, and wavelet transform. The work of Dai et al. focused on extracting time domain and frequency domain features from E-nose signals [14]. Although they attempted to combine the maximum and average values from the time domain with the maximum and average energies obtained from the three-scale wavelet packet decomposition in the frequency domain, this approach was limited to selecting maximum and average energies as representative features, potentially leading to the loss of a considerable amount of useful information. Furthermore, their strategy for feature fusion, simply concatenating features linearly, lacks in-depth interaction analysis, which limits the ability to mine deep connections from limited features. Wang et al. expanded the range of feature extraction, including time domain features such as stable value and area value, coefficients in fitting equations, as well as frequency domain features like total energy and wavelet entropy extracted through wavelet packet decomposition [15]. Additionally, Kou et al. extracted a richer set of features from both the spatial and frequency domains, including characteristics of the spatial domain described by magnitude, difference, derivative, second derivative, integral, slope, and phase features, while applying Fast Fourier transform and wavelet to analyze frequency domain information [1]. Their experimental results revealed the potential of combining different features to enhance the accuracy of specific tasks, with wavelet, slope, and phase features showing significant importance. However, these research treats the extraction and analysis of time domain and frequency domain features as relatively independent, failing to delve into the interaction between time–frequency domain features, thus limiting the model’s ability to handle complex signals. Moreover, the manual feature extraction process overly relies on expert knowledge and experience, potentially overlooking key latent features.

This study proposed a time–frequency attention convolutional neural network (TFA-CNN) for deeply mining the time domain and frequency domain information in E-nose signals and achieved effective integration of these two types of information. The core of this network was that the discrete cosine transform (DCT) was used to replace the traditional Fourier transform (FT) and its inverse transform process. The results could reduce computational cost and avoid the interference of high-frequency noise. Additionally, Maxpooling operation was applied to process time domain signals to reduce time domain noise oscillation and highlight long-term trends. Finally, the model’s ability to analyze E-nose signals has been enhanced by expanding the receptive field (RF) of the convolutional layers, thereby improving the accuracy of predictions.

In deep learning, the generalization ability of a model, which is its performance on unseen data, is considered a key metric for assessing its success. Data augmentation, as a strategy to enhance model performance by increasing the diversity of training samples, has proven effective in the time series domain [16]. In facing the challenge of imbalance among gas samples, Luo et al. adopted the synthetic minority oversampling technique (SMOTE), selecting several samples closely connected to the target sample through the nearest neighbor algorithm, and then using linear interpolation to increase the sample size [17]. Wang et al. further developed this idea by randomly selecting a sample from the training set for augmentation, calculating the Euclidean distance between this sample and other samples on similar labels, and selecting the five closest samples. These selected samples were combined with the original sample, assigning different weights and performing a weighted sum to create new samples, thus achieving the goal of expanding the training set sample size by tenfold [18]. Despite certain successes in data augmentation achieved by the strategies above, limitations remain. These methods rely on linear combinations of samples within the original data set, which may lead to generated data lacking in diversity and failing to cover a broader data distribution.

This work adopted a novel data augmentation strategy, namely signal masking (SM) which introduced changes directly in the data’s feature and temporal dimensions. Masking operations on feature channels can simulate scenarios where partial sensor drift may occur, prompting the model to learn representations that remain effective despite partial information loss. Simultaneously, temporal domain masking, by randomly hiding parts of the time series, forces the model to not only reduce its dependence on irrelevant features, but also enhances its ability to adapt to incomplete or corrupted data that may be encountered in real-world applications. We further introduced a data augmentation strategy called window slicing (WS), which extracted shorter, continuous segments from the E-nose signals. Combining feature channel and temporal domain masking operations with the WS, a comprehensive data augmentation method for E-nose data analysis was proposed. This approach not only addressed the limitations posed by other data augmentation methods, but also enhances the model’s ability to handle brief and incomplete data by introducing content-based variations.

In this paper, we designed and trained a TFA-CNN to achieve gas type discrimination and concentration prediction. Furthermore, we developed a data augmentation method to deal with scenarios lacking reliable and repeatable data. The fully trained model exhibited excellent generalization performance in both tasks. This work provides valuable insights for the further application of deep learning in E-nose systems.

## 2. Related Works

### 2.1. Convolutional Neural Networks

In the application of E-nose systems, numerous pattern recognition algorithms such as the K-nearest neighbors (KNN) [14], support vector machine regression (SVR) [19], and random forest [20] have been reported. These methods have demonstrated their effectiveness in specific applications. However, with the rise of deep learning technology as a new generation of pattern recognition method, its application in the field of E-nose systems has begun to attract researchers’ attention [21]. Specifically, CNNs have been proven to be effective models for processing E-nose signals [22,23,24]. The framework for odor descriptor rating prediction proposed by Guo et al., which integrates Convolutional LSTM layers and regression layers into multiple sibling neural networks, has shown improvements in prediction accuracy [22]. Li et al. introduced an optimization method for detecting mixed gases and reported that the classification accuracy of the CNN model significantly outperforms support vector machine and multi-layer perception when the proportion of the training set becomes small [23].

The concept of the receptive field (RF) is an important tool for understanding deep CNNs [25]. Unlike fully connected networks, neurons in CNNs respond only to a localized area of the input data, which is referred to as the neuron’s RF. The RF provides a mechanism that allows the network to capture both local and global information from the input data at different levels. Typically, the initial convolutional layers of the network have smaller RFs to capture local details of the input data. Subsequent convolutional layers have progressively larger RFs, allowing for the extraction of more complex and abstract features. When processing time series data, the size of the RF determines how “far” the network can “see” in one-dimensional space, affecting its ability to capture longer temporal patterns. Fawaz et al. provided a definition of RF for one-dimensional time series data [26]. With a convolution stride of one and each layer’s filter length being equal to *k_i_*, in a network of depth *d*, the RF can be represented as follows:

(1) $$\mathrm{RF}=\sum_{i=1}^{d}\left(k_{i}-1\right)+1,i\in \left[1,d\right].$$

In computer vision, a larger RF is often required to capture more context for object recognition [25]. Similarly, it can be assumed that detecting a larger region in a one-dimensional time series data requires a larger RF.

Tang et al. discovered that the performance of one-dimensional CNNs is primarily determined by the optimal size of the RF, and it is relatively insensitive to the specific kernel size configurations that make up the RF size. Further findings suggest that if a model could encompass all possible RF sizes, its performance would be similar to that of a model with only the optimal RF size [27]. Therefore, they developed an Omni-scale block (OS-block) that can cover all sizes of RFs.

The architecture of OS-block is a three-layer multicore structure with the first two layers consisting of a set of prime numbers up to *P_k_*, using ℙ*_m_* to denote the set of kernel sizes in the *m*-th layer:

(2) $${\mathbb{P}}_{m}=\left\{\begin{array}{cc} \left\{1,2,3,5,\ldots ,p_{k}\right\}, & m\in \left\{1,2\right\}\\ \left\{1,2\right\}\;\;\;\;, & m=3 \end{array}\right.,$$

Among them, $\left\{1,2,3,5,\ldots ,p_{k}\right\}$ is a set of prime numbers from 2 to *P_k_*, including 1. As described by Equation (1), the set of RF sizes Ω is as follows:

(3) $$\Omega =\left\{{\mathbb{P}}_{1}+{\mathbb{P}}_{2}+{\mathbb{P}}_{3}-2\left|{\mathbb{P}}_{i}\in {\mathbb{P}}_{m},i\in \left\{1,2,3\right\}\right.\right\}.$$

Given that ℙ_1_ and ℙ_2_ are lists of prime numbers with a certain upper limit, according to the Goldbach conjecture, the set $\left\{{\mathbb{P}}_{1}+{\mathbb{P}}_{2}\right\}$ will include all even numbers. With the addition of the third layer kernel sizes of 1 and 2, this design can cover all RF sizes for the length of the time series, thereby capturing features across different time scales. 

### 2.2. Multi-Task Learning

In the application scenarios of E-noses, there is often a need to simultaneously determine gas types and predict concentrations. This typically requires training multiple independent models, thereby increasing the complexity of implementation. For example, to identify gas types and predict concentrations simultaneously, a classifier for gas type identification should be developed firstly, followed by training another regression model for gas concentration prediction. In recent years, the rise of multi-task learning (MTL) techniques has provided a solution to this challenge by sharing hidden layers among various tasks, while preserving independent output layers for each specific task [28]. Moreover, existing studies have shown that learning multiple related tasks simultaneously performs better than learning them independently [29]. Wang et al. have designed a multi-task learning convolutional neural network (MTL-CNN) to carry out three different classification tasks simultaneously: target identification, concentration prediction, and state judgment, achieving an identification accuracy of about 95% for 12 kinds of volatile organic compounds (VOCs) [24]. Research on MTL primarily focuses on exploring more effective mechanisms for parameter sharing, including hard parameter sharing, and soft parameter sharing. Among these, the hard parameter sharing mechanism is widely adopted due to its simplicity in implementation and suitability for handling highly correlated tasks. In practical applications of E-nose systems, where there is sometimes a need to detect both gas components and their concentrations simultaneously, adopting the hard parameter sharing mechanism to simplify the training process is appropriate. 

## 3. Method

In this study, we aim to tackle two tasks: classification and regression. Both tasks share the same training data ${\left(x_{i},y_{i}^{k}\right)}_{i=1}^{n}$, with *n* representing the number of samples, and *x_i_* and *y_i_^k^* representing the sensor’s input data and its corresponding target labels in the *k*-th task, where $k\in \left\{1,2\right\}$ stands for classification and regression tasks, respectively. The training objective of the TFA-CNN framework proposed in this study is to optimize model performance by simultaneously minimizing the loss functions of both tasks:

(4) $$\mathrm{Loss}={\mathrm{Loss}}_{1}\left(f^{1}\left(x\right),y^{1}\right)+{\mathrm{Loss}}_{2}\left(f^{2}\left(x\right),y^{2}\right),$$

where, $f^{k}\left(x\right)$ is the prediction result given by the network based on the input data *x*, and *Loss_k_* represents the loss function for the *k*-th task. 

For the classification task, the loss function uses cross-entropy loss, so the loss can be defined as follows:

(5) $${\mathrm{Loss}}_{1}=-\sum_{i=1}^{n}y_{i}^{1}\log\left(f^{1}\left(x_{i}\right)\right).$$

while for the regression task, mean squared error (MSE) is typically used as the loss function, thus the loss is defined as follows:

(6) $${\mathrm{Loss}}_{2}=\frac{1}{n}\sum_{i=1}^{n}{\left(y_{i}^{2}-{\left(f^{2}\left(x_{i}\right)\right)}^{2}\right)}^{2}.$$

Therefore, the total loss function can be represented as follows:

(7) $$\mathrm{Loss}=\alpha {\mathrm{Loss}}_{1}+\left(1-\alpha \right){\mathrm{Loss}}_{2}.$$

where α is a weight factor, ranging from 0.0 to 1.0, used to balance the importance of the two tasks.

### 3.1. TF-Attention Block

In the field of frequency domain modeling, most methods rely on the process of FT and its inverse transform, which not only adds additional computational burden but also introduces high-frequency noise due to the inherent periodicity of the FT, leading to the Gibbs phenomenon and reducing the accuracy of model predictions. This issue is particularly prominent in applications requiring high-precision predictions, making accurate modeling of time series data an urgent problem to solve. Jiang et al. proposed a frequency-enhanced channel attention mechanism by utilizing the discrete cosine transform (DCT) to replace the commonly used FT-IFT process for frequency extraction. This mechanism integrates frequency domain data into the modeling process, enhancing the model’s ability to extract frequency features [30]. Compared to the FT, DCT not only effectively mitigates the Gibbs phenomenon but also, due to its superior energy concentration properties over the discrete Fourier transform (DFT), becomes a better choice for extracting frequency information from time series.

**Figure 1:**
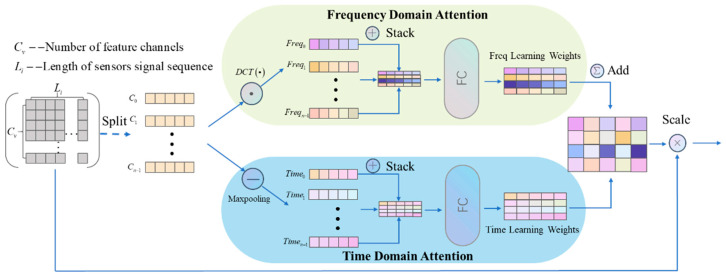
Schematic diagram of TF-attention block. (1) First, use the split operation to divide each multi-channel E-nose signal sequence. (2) Perform DCT and Maxpooling operations on each channel variable separately to generate *Freq_i_* and *Time_i_*. (3) Stack *Freq_i_* and *Time_i_* along the channel dimension to obtain the frequency tensor and time tensor, respectively. (4) Use two fully connected layers to learn from the frequency tensor and time tensor. (5) Fuse the time domain attention and frequency domain attention to form the time–frequency attention *TF_att_*, a step that facilitates the integration of adaptive features from both the temporal and frequency domains. (6) The input sequence is element-wise multiplied by the time–frequency attention to adjust the input data to better utilize its time–frequency domain features.

In order to deeply explore the frequency information embedded in the E-nose signals and extract the implied sequence features more effectively, this study introduces the DCT to convert the time domain information to frequency domain information and proposes a time–frequency attention block (TF-attention block) with the fusion of frequency domain and time domain features. Figure 1 describes the architecture of the TF-attention block. For a given single-channel E-nose signal sequence, the DCT of its length *N* one-dimensional sequence is defined as follows:

(8) $$F\left(k\right)=c\left(k\right)\sum_{i=0}^{N-1}x_{i}\cos\left[\frac{\left(i+0.5\right)\pi }{N}k\right],k=0,1,2,\ldots ,N-1,c\left(k\right)=\left\{\begin{array}{c} \sqrt{\frac{1}{N}},k=0\\ \sqrt{\frac{2}{N}},k\neq 0 \end{array}\right..$$

where *x_i_* is the sequence of E-nose signals for a single channel, and $F\left(k\right)$ is the transformed sequence.

The process of TF-attention block is as follows:

(9) $$c_{0},c_{1},\ldots ,c_{n-1}=\mathrm{Split}\left(x\right),$$

(10) $${\mathrm{Freq}}_{i}={\mathrm{DCT}}_{i}\left(c_{i}\right),$$

(11) $${\mathrm{Time}}_{i}={\mathrm{MaxPooling}}_{i}\left(c_{i}\right),$$

(12) $$\mathrm{Freq}=\mathrm{Stack}\left({\mathrm{Freq}}_{0},{\mathrm{Freq}}_{1},\ldots ,{\mathrm{Freq}}_{n-1}\right),$$

(13) $$\mathrm{Time}=\mathrm{Stack}\left({\mathrm{Time}}_{0},{\mathrm{Time}}_{1},\ldots ,{\mathrm{Time}}_{n-1}\right),$$

(14) $$TF_{att}=\mathrm{Add}\left({\mathrm{Fc}}_{1}\left(Freq\right),{\mathrm{Fc}}_{2}\left(\mathrm{Time}\right)\right),$$

(15) $$x_{out}=TF_{att}\cdot x.$$

First, the E-nose input signal $x\in R^{n\times L}$ is segmented along the channel dimension into n independent sensor channel variables $c_{i}\in R^{1\times L},i\in \left\{0,1,2,\ldots ,n-1\right\}$. This step is based on the premise that each sensor may have different characteristics as well as unique frequency and time information, thereby ensuring that individualized processing can be carried out for each channel. For each channel variable obtained from the segmentation, we first apply the DCT to convert the time domain signal into a frequency domain signal. Subsequently, each frequency channel vector is stacked along the channel dimension to form a frequency tensor $\mathrm{Freq}\in R^{n\times L}$. For the time domain signal, Maxpooling technology is employed to mitigate noise oscillations and highlight long-term trends, and then further stacked along the channel dimension to form a time tensor $\mathrm{Time}\in R^{n\times L}$. 

Following this, we processed the frequency tensor and time tensor separately through two independent fully connected networks to capture the time–frequency dependencies among different channels. Then, we fused the generated time domain and frequency domain attentions to form a comprehensive time–frequency attention $TF_{att}\in R^{n\times L}$. Finally, the original signal input is element-wise multiplied by the time–frequency attention, thereby adjusting the input data to better suit its time–frequency features. By introducing the time–frequency attention mechanism, our model not only achieves fine-grained adaptive feature extraction in both the time and frequency domains but also effectively enhances the model’s capability in fusing features from the frequency and time domains. Ultimately, the effectiveness of the proposed method through ablation experiments has been demonstrated.

In this study, the TF-attention block forms a base feature extraction framework with the OS-block; the OS-block is placed after the TF-attention block to obtain the optimal RF size for the time–frequency features. 

### 3.2. Data Augmentation

In this section, the data augmentation strategies implemented for E-nose signal were described. Given that E-nose sensors may experience drift phenomena due to environmental factors in practical applications, it is important to note that drift might not occur in all sensors of the array. Firstly, considering that sensor drift could lead to more severe feature confusion than the loss of information caused by the absence of some sensor signals, a channel masking technique was adopted. Specifically, this technique randomly selects $m_{v}$ times to mask [0, m) channels, where $0<m_{v},\;m<n$, *n* represents the total number of channels. Additionally, by acknowledging the potential presence of abundant redundant or irrelevant feature information in E-nose signals, such as the signals before and after adsorption is smooth so there are fewer features, a time masking strategy was proposed. This involves t consecutive time steps $\left[t_{0},\;t_{0}+t\right)$ that are masked, where *t* is randomly chosen from a uniform distribution ranging from 0 to the time masking parameter *T*, *t*_0_ is chosen from $\left[0,\;L-t\right],\;t<L$ and *L* represents the length of the E-nose signals.

Secondly, considering the relationship between data length and the amount of feature information, overly lengthy input data can not only extend training duration but also potentially lead the model to learn redundant or irrelevant information, thereby affecting training efficiency and the model’s generalizability. Therefore, the WS strategy was used to focus on local segments of the E-nose signals for effectively capturing short-term dependencies in the data, which involved extracting slices of a specific length from the E-nose signals and incorporating them into the training set, ensuring each slice retains the original label. To maintain consistency in the lengths of the data segments, it is necessary to upsample the sliced data to match the original sequence length. Methods similar to WS have been applied in related studies of E-nose systems [22,31].

By implementing SM to simulate sensor loss and reduce the interference of invalid information, along with adopting WS strategy to accommodate the model’s needs for analyzing local segments, our method not only enhances the model’s ability to learn hidden patterns in the data but also improves its robustness to incomplete information. Consequently, this approach enhances the diversity of the data while also boosting the model’s generalization performance. Table 1 presents the data augmentation parameters adopted in this study.

## 4. Experiment

To ensure consistency in the model training and testing processes, all algorithms in this study were implemented in an Integrated Development Environment (IDE) equipped with Python 3.8 (PyCharm 2023.1.3, Community Edition). The programs were run on a Windows 11 operating system (CPU: Intel Core i7-12700F (Santa Clara, CA, USA), GPU: NVIDIA RTX 3060 (Santa Clara, CA, USA), RAM: 64 GB). To accelerate the deep learning training process, GPU parallel computing was utilized, employing the GPU version of Tensorflow (V2.7.0) as the main computational tool. Regarding the model training parameter settings, a batch size of 32, a number of epochs of 200, and a learning rate of 0.001 were selected. Moreover, “Adam” was used as the optimizer, a widely adopted optimization technique in the field of deep neural networks.

### 4.1. Network Architecture

**Figure 2:**
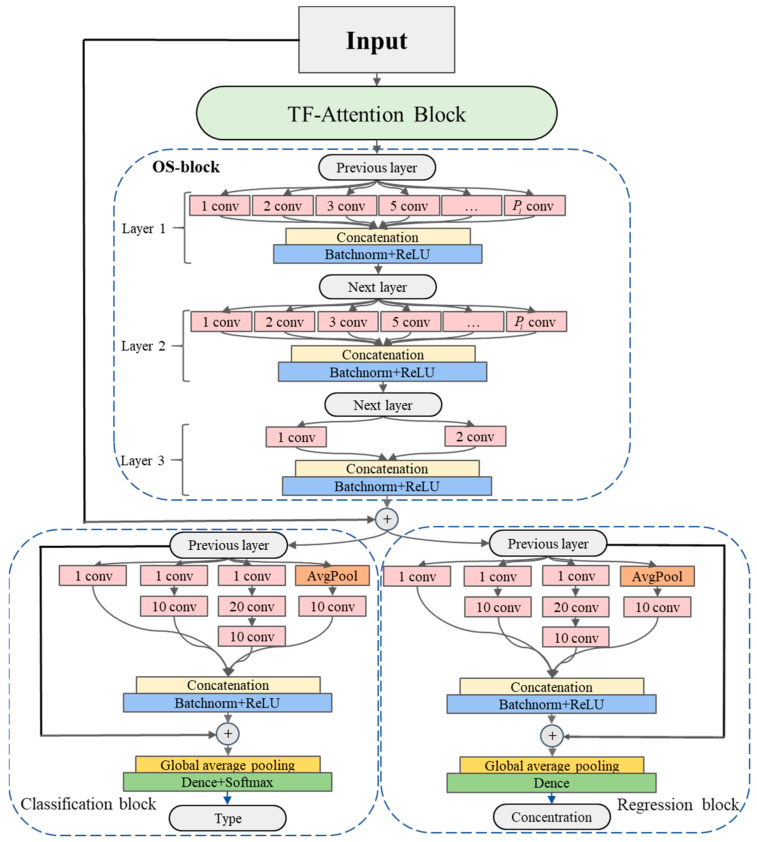
The TFA-CNN model based on time–frequency attention. The model is divided into three main parts: (1) The initial input E-nose signal data first flows through the TF-attention block, effectively integrating the time domain and frequency domain features of the data. (2) Subsequently, the data enters a three-layer OS-block, which promotes the capture of richer features by expanding the RF. (3) Finally, the data is transmitted to the Classification block and Regression block, respectively. These two blocks focus on the specific feature learning of their respective tasks and output results related to the tasks.

As shown in Figure 2, the base feature learning layer is comprised of the TF-attention block and OS-block, achieving shared feature extraction from the original data. Building on this foundation, to address specific regression and classification tasks, subsequent network blocks designed for these specific tasks were employed. These include parallel stacked Classification and Regression blocks, enabling simultaneous execution of gas type classification and gas concentration prediction tasks. Both blocks have similar structures, each processing the input tensor in parallel four times. The first three processing steps utilize convolution operations with varying numbers of layers. The first layer of convolution uses filters with a length and stride of 1, reducing the input sequence from *T* dimensions to *m* dimensions. This dimensionality reduction significantly simplifies the model structure and mitigates overfitting issues in small data sets. By employing multi-layer convolution, the RF for specific tasks is gradually expanded, while different lengths of filters are used to extract multi-resolution features. The fourth processing step involves average pooling to reduce the model’s sensitivity to minor perturbations, followed by a convolution layer to further reduce data dimensions. Finally, the outputs from each parallel convolution processing are concatenated to form the final output.

At this stage, despite the processing of feature data, its dimensions remain large, which not only increases computation time but may also lead to overfitting. The introduction of a global average pooling layer effectively reduces the size of the feature data. Finally, through a fully connected layer, the extracted features are mapped to the sample label space, obtaining outputs for both classification and regression tasks. To assess network performance and optimize the model parameters, this study employed a five-fold cross validation method. By training on different subsets of the training data, this approach provides more robust evaluation results and efficient parameter selection results.

### 4.2. Evaluation Metrics

To evaluate the predictive performance of the model, this study uses accuracy as the assessment metric for the classification task. Meanwhile, for regression task, MAE, the *r*, and the *R*^2^ are introduced to measure the model’s performance:

(16) $$Accuracy=\frac{Number\;of\;correctly\;predicted\;samples}{Total\;number\;of\;samples},$$

(17) $$\mathrm{MAE}=\frac{1}{n}\sum_{i=1}^{n}\left|y_{i}-{\hat{y}}_{i}\right|,$$

(18) $$r=\frac{\sum_{i=1}^{n}\left(y_{i}-\bar{y}\right)\left({\hat{y}}_{i}-\bar{\hat{y}}\right)}{\sqrt{\sum_{i=1}^{n}{\left(y_{i}-\bar{y}\right)}^{2}}\sqrt{\sum_{i=1}^{n}{\left({\hat{y}}_{i}-\bar{\hat{y}}\right)}^{2}}},$$

(19) $$R^{2}=1-\frac{\sum_{i=1}^{n}{\left(y_{i}-{\hat{y}}_{i}\right)}^{2}}{\sum_{i=1}^{n}{\left(y_{i}-\bar{y}\right)}^{2}}.$$

where ${\hat{y}}_{i}$ represents the model’s prediction for the *i*-th sample, and $y_{i}$ represents the corresponding true value. $\bar{y}$ and $\bar{\hat{y}}$, respectively, represent the mean of the true values and the mean of the predicted values. The highest value for accuracy is 100%, indicating that all type recognitions are correct. A lower MAE value indicates a lower level of error in the model, meaning higher prediction accuracy. At the same time, when the *r* and the *R*^2^ values are closer to 1, it signifies a higher degree of model fit, indicating superior predictive performance.

### 4.3. Dataset

In this study, the four gas sensors used were independently prepared by our laboratory, manufactured using the radio frequency magnetron sputtering, with specific sputtering parameters detailed in Table 2. These sensors had different In_2_O_3_/SnO_2_ ratios (wt%) set at 80:20, 85:15, and 90:10, as well as the tin oxide (SnO_2_) sensor. The choice of these four different sensors aims to utilize their cross-selectivity, thereby enhancing the capability to identify specific gases. 

Five target gases were collected: ethanol, acetone, methanol, nitrogen dioxide and sulfur dioxide. In the setup for each individual experiment, the concentration range for each target gas was set from 7.5 ppm to 75.0 ppm, increasing in steps of 7.5 ppm, with each experiment independently repeated five times.

To accurately capture the response and recovery behavior of the gas sensors, standardized time intervals were employed. The specific process is as follows. First, the sensor array was left to stabilize in the air for 5 min after turning on the testing system. Then, the protective cover was closed within 10 s, and a specific concentration of the target gas was introduced, maintaining this state for 60 s. Subsequently, the protective cover was opened, and nitrogen gas was used to purge the inside of the protective cover for 1 min to remove residual gas. Finally, after a 4 min interval, the next round of data collection began. When completing the collection for a certain gas, the experiment was paused for over 24 h to mitigate sensor fatigue or damage caused by frequent testing; then, collection continued for the next gas type. Following this procedure, all collection work in this study lasted about half a month, ultimately accumulating over 50,000 valid data points, as detailed in Table 3.

Considering the rapid response and recovery characteristics exhibited by sensors in gas detection, which generally allow for the detection of gas responses to be completed within about 3 and a half minutes, this study utilized only the response values within the first 210 s of each detection as the input sequence. Ultimately, a total of 250 data samples were obtained.

### 4.4. Discussion

The experimental data were divided into two parts: training data and testing data, with a ratio of 4:1. By using the data augmentation parameters from Table 1, the training data was expanded tenfold, from 200 training samples to 2000 samples. The training data used 5-fold cross validation to search for the optimal model parameters, while the testing data was reserved for final evaluation. To assess the performance of the base feature learning layer composed of the TF-attention block and OS-block in classification and regression tasks, two types of CNNs for comparison were introduced. These include the RTFA-CNN, which focused on the concentration prediction task by removing the classification task branch, and the CTFA-CNN, dedicated to the gas type classification task by eliminating the regression task branch. A fully convolutional network (FCN) is also introduced in this study for comparison. FCN was proposed by Wang et al. for processing time series classification tasks [32], which replaced the fully connected layer in traditional CNNs with convolutional layers. It mainly consisted of three convolutional blocks, with batch normalization and ReLU activation functions attached after each convolutional block, and a global average pooling layer applied after the last convolutional block, which is then connected to a softmax classifier for time series data classification. It was the most accurate deep learning model in the evaluation of 12 multivariate time series classification datasets [33]. In addition, Tan et al. extended the application of the FCN network to time series regression analysis by replacing the softmax function with a linear activation function, enabling the model to predict continuous values and demonstrating leading performance on multiple datasets [34].The FCN model used the network structure and parameter configurations proposed by Tan et al. [34]. To further enrich the comparison baseline, three widely used pattern recognition algorithms were also compared, including extreme gradient boosting (XGBoost), random forest, and SVR. For these three algorithms, a grid search method was used to adjust hyperparameters, aiming to find the optimal parameter configuration. All algorithms were implemented on the same computing platform as TFA-CNN.

**Figure 3:**
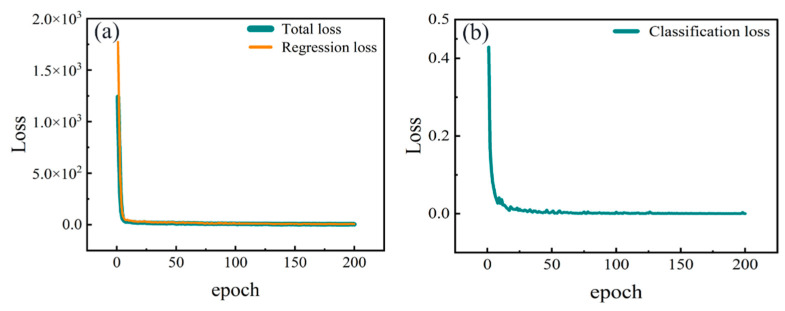
The decline in the loss of the TFA-CNN during the training process: (**a**) regression loss, (**b**) classification loss.

The final training model based on the TFA-CNN demonstrated excellent stability and generalizability. After 200 epochs of training, the model’s total loss was stabilized, with no overfitting observed. The variation curves of regression and classification losses are, respectively, illustrated in Figure 3a,b.

**Figure 4:**
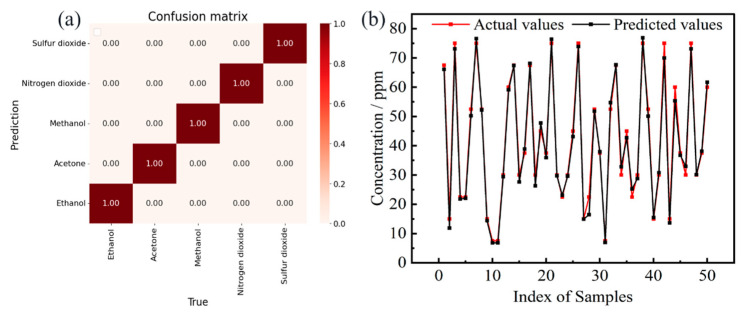
Results of the TFA-CNN on the testing set: (**a**) confusion matrix for type recognition, (**b**) actual and predicted values of concentration.

The confusion matrix for type identification, as shown in Figure 4a, indicated that the accuracy of the classification task for the five target gases reached 100%. Figure 4b displayed the comparison between actual and predicted concentrations for 50 randomly selected test samples. Experimental results demonstrated that the model exhibited stable generalization capability in concentration prediction tasks. However, the error rate in low concentration intervals increases compared to higher concentrations. This could be due to the sensor’s response in low concentration ranges not being as significant or linear as under high concentration conditions, resulting in a relatively larger variance in sensor characteristics (such as resistance or voltage changes). Such an expansion of characteristic intervals may introduce uncertainty to the model’s prediction accuracy. Furthermore, sample collection at low concentrations might increase the randomness of sampling bias due to experimental errors, thereby amplifying the differences between sampling results. The results revealed that there was a good consistency between the predicted values and actual values by the TFA-CNN model, indicating that the MTL architecture was extremely effective for gas concentration prediction.

Table 4 provides a summary of statistical data for seven models. The results highlight that the TFA-CNN, RTFA-CNN and CTFA-CNN models exhibit excellent performance in combined classification and regression tasks, the individual regression and classification tasks, respectively. For the TFA-CNN model (The results with bold font in Table 4), the classification accuracy reached 100%, the *R*^2^ score for regression tasks was as high as 0.99, the *r* was 0.99, and the MAE was 1.54 ppm. 

Overall, compared to other common pattern recognition algorithms, TFA-CNN not only demonstrated more robust learning capabilities but also offered a more flexible design pattern, outperforming other models in overall performance.

### 4.5. Ablation Experiment

To systematically evaluate the contribution of the proposed TF-attention block and data augmentation strategies to model performance, this study designed a series of ablation experiments. These experiments aimed to verify the effectiveness of each module in enhancing the model’s learning ability. 

Comparative experiments were conducted to analyze the changes in the performance of the model after removing the TF-attention block, removing the frequency attention alone, or removing the time attention alone. The results of the experiments are organized in Table 5. The results showed that the absence of any form of attention mechanism leads to a decrease in model performance, with the removal of the TF-attention block having the most significant effect. The TF-attention block, by effectively weighting the input signals in both time and frequency domains, enables the model to focus more on information beneficial to the prediction task, thus improving the model’s accuracy and generalization ability. 

The specific contribution of each data augmentation method to the model performance is assessed by removing any of the three methods, channel masking, time masking and window slicing, respectively. The experimental results are shown in Table 6, where the generalization performance of the model decreased when any of the methods are absent. Using any two methods had a positive impact on the model’s generalization performance with similar enhancement, and it can be hypothesized that each method can enhance the diversity of the samples in different feature dimensions and optimize the model’s performance in its specific dimensions. When all three methods are applied simultaneously, the diversity of the dataset is maximized, thus achieving the best generalization effect.

When the data augmentation strategy was not used, the model’s MAE increased from 1.54 ppm to 3.13 ppm, the *R*^2^ decreased from 0.990 to 0.952, and the *r* dropped from 0.996 to 0.980. This result verified the practical benefits of the proposed data augmentation strategy in improving model stability and prediction accuracy.

### 4.6. Independent Validation Experiment

To evaluate the model’s performance on unseen data, 45 sets of data were recollected to thoroughly analyze the model’s generalization ability. The specific types of gases and concentration ranges involved were detailed in Table 7. The training set comprises 30 sets of data, covering three types of gases at five different concentration levels, with each concentration level spaced by 10 ppm and sampled twice. The testing set included 15 sets of data, covering three types of gases at five different concentration levels, with each concentration level spaced by 10 ppm and sampled once, and the concentration levels in the training and testing sets did not overlap. 

This validation experiment was conducted through fine-tuning the pre-trained TFA-CNN model, whose parameters were derived from training on five gases. Specific adjustments included removing the last classification layer in the regression task and adjusting this layer from the original five neurons to three to accommodate the classification task for three types of gases, while the configurations of other layers remained unchanged. The fine-tuning process was conducted in the same training environment as the TFA-CNN, with training parameters including a batch size of 8, a number of epochs of 200, and a learning rate set at 0.001.

**Figure 5:**
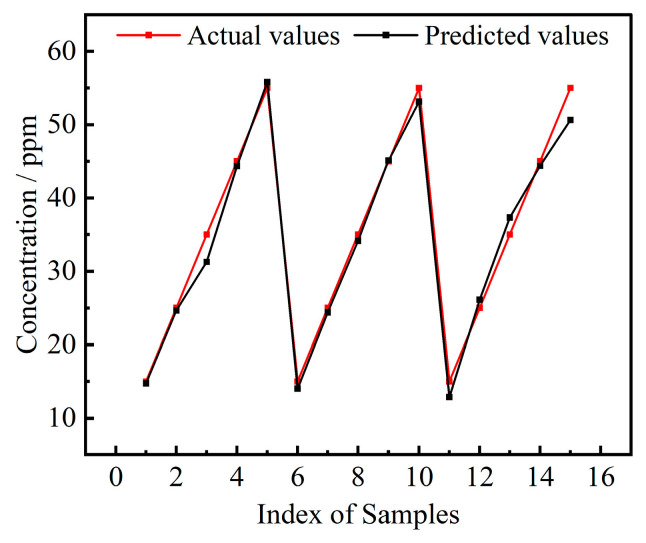
Actual and predicted values in the testing set of the independent validation experiment.

Typically, a smaller data set size increases the risk of the model encountering overfitting. As shown in Figure 5, the fine-tuned TFA-CNN model’s concentration prediction results on the test set effectively demonstrate the efficacy of data augmentation techniques in reducing the risk of overfitting. Even with only 30 sets of training samples collected, data augmentation methods could help the model obtain more robust prediction results. 

Table 8 summarizes the performance of the seven models in the experiment. It further validates that the TFA-CNN model (The results with bold font in Table 8), after undergoing training on a larger data set and subsequent fine-tuning, could still effectively grasp the response mechanisms of gas-sensitive sensors to various types of gases and maintain outstanding performance when faced with new data challenges. 

Additionally, when faced with a testing set containing unseen concentration ranges, traditional pattern recognition methods such as random forest, SVR, and XGBoost demonstrated significantly poorer generalization abilities compared to deep learning models, with MAE all exceeding 3.34 ppm. This result further validates the effectiveness of deep learning models in feature learning.

## 5. Conclusions

In this study, we proposed and validated a time–frequency attention convolutional neural network, specifically designed for gas type identification and concentration prediction tasks. This model integrated a time–frequency attention mechanism with deep convolutional neural networks, enabling the network to capture and merge temporal and frequency domain information in E-nose signals at the base feature learning layer. By widening the RF of convolutional layers, the model’s capability to capture gas signal features was enhanced, thereby improving gas detection and concentration estimation performance.

Experimental results indicated that the TFA-CNN model accurately predicted the gas categories and their concentrations. The accuracy of the model reached 100%, the coefficient of determination of 0.99, the Pearson correlation coefficient was also 0.99, and the MAE was only 1.54 ppm, which is significantly better than other models. Furthermore, ablation experiments validated the effectiveness of this proposed TF-attention block and data augmentation methods. The model also performed well in independent validation experiments, obtaining accurate results on the testing set of unseen concentration ranges, with a classification accuracy of 100%, the coefficient of determination of 0.98, the Pearson correlation coefficient of 0.99, and the MAE of only 1.39 ppm, further verifying the model’s ability to learn the patterns of gas responses.

## Figures and Tables

**Table 1:** Data augmentation parameters.

	WS		SM			
Data augmentation	Ratio	Number	*m*	*m_v_*	*t*	*m_t_*
	0.9	3	2	3	10	3

(Where *m_v_* and *m_t_*, respectively, indicate the number of applications of channel masking and time masking, *m* denotes the number of channels masked in each application, and *t* specifies the duration of each masking operation along the time dimension. “Ratio” reflects the proportion of each slice relative to the original data length, while “Number” denotes the total number of slicing operations performed).

**Table 2:** Parameters of radio frequency magnetron sputtering.

Labels	In_2_O_3_:SnO_2_ (wt%)	Sputtering Time (min)	Sputtering Temperature (°C)	RF Power (W)	Ar:O_2_
0100	00:100	4	300	300	20:0
9010	90:10				
8515	85:15				
8020	80:20				

**Table 3:** Gas concentration range and quantity.

Type	Days	Repetition	Concentration Range (ppm)	Number
Ethanol	1–3	5	7.5–75.0	10
Acetone	4–6	5	7.5–75.0	10
Methanol	7–9	5	7.5–75.0	10
Nitrogen dioxide	10–12	5	7.5–75.0	10
Sulfur dioxide	13–15	5	7.5–75.0	10

**Table 4:** Performance of the seven models on the dataset.

Model	Classification Task	Regression Task		
	Accuracy	MAE	*R* ^2^	*r*
TFA-CNN	**100%**	**1.542**	**0.990**	**0.996**
RTFA-CNN	-	1.818	0.987	0.994
CTFA-CNN	100%	-	-	-
FCN	-	2.597	0.971	0.989
Random forest	-	3.469	0.949	0.975
SVR	-	3.438	0.956	0.979
XGBoost	-	3.727	0.943	0.972

**Table 5:** Results of time–frequency attention ablation experiments (✗ represents No; ✓ represents Yes).

Frequency Attention	Time Attention	Data Augmentations	Classification Task	Regression Task		
			Accuracy	MAE	*R* ^2^	*r*
✗	✗	✓	100%	2.222	0.979	0.993
✗	✓	✓	100%	1.875	0.984	0.994
✓	✗	✓	100%	1.767	0.986	0.994

**Table 6:** Results of data augmentation ablation experiments (✗ represents No; ✓ represents Yes).

Channel Masking	Time Masking	Window Slicing	Classification Task	Classification Task		
			Accuracy	MAE	*R* ^2^	*r*
✓	✗	✓	100%	1.853	0.983	0.992
✗	✓	✓	100%	1.803	0.985	0.993
✓	✓	✗	100%	1.837	0.985	0.993
✗	✗	✗	100%	3.134	0.952	0.980

**Table 7:** The range and quantity of gas concentrations in the independent validation experiment.

Type	Repetition	Concentration Range (ppm)	Number	Mode
Ethanol	2	10.0–50.0	5	Train
	1	15.0–55.0	5	Test
Acetone	2	10.0–50.0	5	Train
	1	15.0–55.0	5	Test
Methanol	2	10.0–50.0	5	Train
	1	15.0–55.0	5	Test

**Table 8:** Performance of the seven models on the independent validation dataset.

Model	Classification Task	Regression Task		
	Accuracy	MAE	*R* ^2^	*r*
Fine-tuned TFA-CNN	**100%**	**1.386**	**0.983**	**0.993**
RTFA-CNN	-	2.182	0.956	0.980
CTFA-CNN	100%	-	-	-
FCN	-	2.414	0.949	0.988
Random forest	-	6.940	0.525	0.779
SVR	-	3.345	0.915	0.970
XGBoost	-	8.337	0.447	0.711

## Data Availability

The data presented in this study are available on request from the corresponding author.
